# Relationships Between Fear of Cancer Recurrence, Unmet Healthcare Needs, and Quality of Life Among Thai Breast Cancer Survivors Post-Treatment

**DOI:** 10.3390/healthcare14020226

**Published:** 2026-01-16

**Authors:** Patcharaporn Pichetsopon, Piyawan Pokpalagon, Nipaporn Butsing

**Affiliations:** 1 Master of Nursing Science in Adult and Gerontological Nursing (Candidate), Ramathibodi School of Nursing, Faculty of Medicine Ramathibodi Hospital, Mahidol University, Bangkok 10400, Thailand; patcharaporn.pic@student.mahidol.ac.th; 2 Ramathibodi School of Nursing, Faculty of Medicine Ramathibodi Hospital, Mahidol University, Bangkok 10400, Thailand; nipaporn.but@mahidol.ac.th

**Keywords:** fear of cancer recurrence, health services needs and demand, quality of life, breast cancer survivors, survivorship care plans

## Abstract

**Highlights:**

**What are the main findings?**

- Fear of cancer recurrence and unmet healthcare needs were significantly associated with poorer overall quality of life among Thai breast cancer survivors after treatment.
- No significant association was found between fear of cancer recurrence and unmet healthcare needs, suggesting distinct psychosocial processes in survivorship.

**What are the implications of the main findings?**

- Survivorship care should integrate psychological and supportive care services to address fear of cancer recurrence and unmet healthcare needs, with attention to improving access to psychosocial care and survivorship education.
- Healthcare systems and survivorship programs may reduce disparities by implementing culturally tailored follow-up services and routine psychological assessment during the post-treatment phase.

**Abstract:**

**Purpose**: This study examined the relationships among fear of cancer recurrence (FCR), unmet healthcare needs, and quality of life (QOL) among breast cancer survivors post-treatment, particularly within the Thai cultural and healthcare context, where limited research has been conducted. **Methods**: A cross-sectional descriptive correlational design with purposive sampling was used. A total of 122 breast cancer survivors, 1–5 years prior, were recruited from the Breast Clinic and Chemotherapy Unit at the National Cancer Institute. Instruments included a demographic questionnaire, the FCR Inventory Short Form, the Cancer Survivors’ Unmet Needs measure, and the EORTC QOL-C30 with the breast cancer module (QLQ-BR23). Cronbach’s α ranged from 0.82 to 0.92. Data were analyzed using descriptive statistics, Spearman’s rank correlation, and Pearson’s correlation coefficient. **Results:** Participants reported moderate levels of FCR (M = 13.39, SD = 4.50), low unmet healthcare needs (M = 25.63, SD = 14.82), and moderate overall QOL (M = 54.82, SD = 0.22). FCR was negatively correlated with overall QOL (r = −0.248, *p* <0.01) and functional QOL (r = −0.242, *p* < 0.01). Unmet healthcare needs were also negatively correlated with overall QOL (r = −0.261, *p* < 0.01). Multiple linear regression analysis revealed that both FCR and unmet healthcare needs had a significantly negative relationship with overall QOL (*p* < 0.05). **Conclusions**: FCR and unmet healthcare needs independently impair QOL among breast cancer survivors. Early, culturally appropriate survivorship care in Asian contexts is essential to address these needs and improve QOL.

## 1. Introduction

Breast cancer remains the most common malignancy among women globally, posing a significant public health concern. In 2022, the World Health Organization reported approximately 2.3 million new cases and 670,000 deaths worldwide [1]. In Thailand, data from the Ministry of Public Health in 2020 indicated around 18,000 new female breast cancer cases annually, with approximately 4800 deaths [2]. Risk factors for breast cancer include age, family history, hormonal influences, and lifestyle behaviors, all of which are associated with disease incidence [3]. While treatment outcomes have improved, survivorship is now a distinct phase requiring attention to psychosocial and supportive care, not only survival statistics. Survivorship following treatment completion (typically 1 to 5 years post-treatment), which reflects the extended survivorship phases, is a critical period marked by diminished healthcare support and ongoing psychosocial and physical challenges [3,4]. Reported recurrence within five years, ranging from 10 to 30% reflects substantial variation across cancer stages, treatment modalities, and healthcare contexts, and therefore should be interpreted cautiously [5,6]. Nevertheless, many survivors continue to experience long-term treatment-related side effects that may adversely affect QOL [7,8].

QOL refers to an individual’s perception of how illness or treatment affects their daily functioning and overall well-being. It encompasses functional status, symptom burden, and an evaluation of health [9]. Among breast cancer survivors, psychological QOL is often significantly affected, particularly in those diagnosed three years earlier [10]. Research suggests that psychological QOL tends to decline in the first year after treatment but improves over time, possibly due to adaptation to symptoms and side effects of treatment [11,12]. However, the overall picture remains unclear, and more research is needed to fully understand how QOL changes during the post-treatment phase. In Thailand, longitudinal and comparative studies examining QOL during this phase are limited [13]. Cultural factors, especially family caregiving, close social ties, and collective decision-making, may influence survivorship experiences differently from Western populations [14]. Understanding these cultural influences is crucial for developing culturally appropriate survivorship care for Thai breast cancer survivors.

Commonly, breast cancer survivors experience unmet healthcare needs, which contribute to uncertainty regarding their illness and recovery [15]. A review of the literature indicates that unmet needs differ across regions. In Western countries, they mainly involve psychological support and health system information, though prevalence may be lower due to structured survivorship care [16,17]. In Asian settings, particularly resource-limited regions, unmet needs are more frequent and severe, especially in information and psychosocial support [18]. In Thailand, survivors report varied unmet needs across physical, psychological, and social domains [19]. Individual-level factors such as resilience, perceived stress, and social support, together with culturally embedded family caregiving norms, may influence how unmet healthcare needs affect psychological well-being and QOL [19]. Nevertheless, empirical evidence examining these relationships in the Thai context remains limited.

Fear of cancer recurrence (FCR) is another significant psychological challenge that often arises shortly after treatment and can persist for several years. FCR can lead to anxiety, distress, and decreased daily functioning, which in turn lowers QOL [20,21,22]. Despite its relevance, there are few studies in Thailand exploring the relationship between FCR and QOL among post-treatment breast cancer survivors. This gap highlights the need to generate culturally specific evidence, since Asian survivors may cope with recurrence fears differently due to stronger family involvement and spiritual beliefs, as compared to Western cohorts.

To guide the present study, Mishel’s Uncertainty in Illness Theory provides a relevant conceptual framework [15]. The theory posits that uncertainty in illness is a central stressor in illness experiences, influencing individuals’ adaptation and quality of life. In this context, FCR serves as a trigger for illness-related uncertainty, while unmet healthcare needs may exacerbate uncertainty by limiting access to information, reassurance, and supportive care. Previous research suggests that illness uncertainty may negatively affect QOL both directly and indirectly through increased psychosocial distress [23,24]. However, empirical evidence testing these theoretical relationships among breast cancer survivors in Thailand is limited.

Therefore, this study aims to examine the relationships among FCR, unmet healthcare needs, and QOL among Thai breast cancer survivors during the post-treatment period. Based on Mishel’s theory, it is hypothesized that higher levels of FCR and greater unmet healthcare needs will be associated with poorer QOL. Findings from this study are expected to contribute to the development of culturally appropriate survivorship care interventions for Thai breast cancer survivors.

## 2. Materials and Methods

### 2.1. Study Design

This study employed a cross-sectional descriptive correlational study and was conducted at the Outpatient Department of the Breast Clinic and Chemotherapy Unit of the National Cancer Institute, Bangkok, Thailand, from December 2024 to February 2025.

### 2.2. Samples/Participants

The sample size was determined based on Cohen’s (1988) [25] effect size estimation and the smallest correlation coefficient reported in prior studies (r = 0.263) [26]. The G*Power software version 3.1.9.7 [27] was used with a significance level (α) of 0.05, statistical power of 0.80, and a two-tailed test. The analysis indicated that a minimum of 111 participants was required. To account for potential sample loss or incomplete responses, the sample size was increased by 10% [28], resulting in 122 participants.

Participants were recruited using purposive sampling from a single tertiary cancer hospital, which allowed access to a defined population of post-treatment breast cancer survivors. However, purposive sampling from a single tertiary hospital may limit the generalizability of the findings. The inclusion criteria were as follows: (1) women aged 18 years or older; for those ≥60 years, screening with the Six-Item Cognitive Impairment Test (6CIT) required scores between 0 and 7; (2) a diagnosis of stage I–III primary breast cancer without metastasis; and (3) completion of primary treatment including surgery, and/or radiotherapy, and/or chemotherapy within 1–5 years prior to study enrollment. All participants were able to communicate in Thai and provided written informed consent. Participants were excluded if they had recurrent or metastatic breast cancer, severe clinical conditions (e.g., dyspnea, extreme fatigue), or comorbidities likely to affect quality of life, such as other cancers, hemiplegia or paralysis, heart disease, chronic kidney disease, or other serious medical conditions.

### 2.3. Instruments

The instruments used in this study were divided into screening and data collection instruments.

#### 2.3.1. 6-Item Cognitive Impairment Test (6CIT-Thai)

The 6CIT was developed by Brook and Bullock [29] and validated in Thai by Aree-Ue and Youngcharoen [30]. It has six items covering three cognitive domains. Scores range from 0 to 28, with scores ≤ 7 indicating no cognitive impairment. The Thai version demonstrates test–retest reliability (r = 0.64, *p* < 0.001) [30].

#### 2.3.2. Demographic and Clinical Information Questionnaire

A researcher-developed questionnaire was used to collect sociodemographic and clinical characteristics.

#### 2.3.3. Fear of Cancer Recurrence Inventory-Short Form (FCRI-SF)

The FCRI-SF is a 9-item unidimensional scale derived from the original 42-item FCRI to assess fear of cancer recurrence [31]. It demonstrated strong criterion validity through correlation with the full FCRI (r = 0.84). Construct validity was further supported by moderate-to-strong correlations with other measures of fear of cancer recurrence (r = 0.59–0.77) and with theoretically related psychological constructs, such as anxiety and distress (r = 0.34–0.62), confirming that the instrument effectively captures the core construct of fear of cancer recurrence. The Thai version of the FCRI-SF, translated by Janthathai et al. [32], uses a 5-point Likert scale, with total scores ranging from 0 to 36 and categorized as low (0–12), moderate (13–21), and high (22–36) levels of fear. Internal consistency in the present study was good (Cronbach’s α = 0.89).

#### 2.3.4. Cancer Survivors’ Unmet Needs Measure (CaSUN)

The CaSUN was developed by Hodgkinson et al. [33]. It contains 35 items scored on a 4-point Likert scale (0–3), with total scores ranging from 0 to 105. Higher scores indicate greater unmet needs. The Thai version was adapted by Pongthavornkamol et al. [34] through a standard translation-and-back translation process. Construct validity was evaluated for the full 5-factor model (28 items). The model showed good fit indices: χ^2^/df = 1.83, CFI = 0.901, SRMR = 0.074, and RMSEA = 0.076, indicating good overall fit and construct validity. All five factors—Existential Survivorship, Comprehensive Care, Information, Quality of Life, and Relationships—had factor loadings ≥0.3. It demonstrated excellent internal consistency, with Cronbach’s α ranging from 0.96 to 0.97 [33,34].

#### 2.3.5. European Organisation for Research and Treatment of Cancer Quality of Life Questionnaire Core 30 (EORTC QLQ-C30) and Breast Cancer–Specific Module (QLQ-BR23)

These two modules were developed to assess QOL among cancer patients [35,36]. The QLQ-C30 (version 3.0) comprises 28 items measuring overall health status, functional, and symptom scales. QLQ-BR23 contains 23 breast-cancer-specific items. Most items use a 4-point Likert scale, while two global health items use a 7-point scale. The validated Thai version shows high overall reliability (α = 0.88), with individual subscale reliabilities of 0.82 (role function), 0.74 (complications), and 0.86 (self-assessment of health status and QOL) [37]. After obtaining the scores from the participants’ responses, the scores were calculated to classify the level of quality of life according to the criteria. The scores range from 0 to 100. Higher scores on the functional ability domain indicate normal functioning and good quality of life. Higher scores on the symptom domain indicate greater abnormalities, while higher scores on the overall health status domain represent better QOL.

### 2.4. Ethical Considerations

This study was conducted in accordance with the ethical standards of the Declaration of Helsinki (revised 2013) and approved by the Institutional Review Board of the Faculty of Medicine, Ramathibodi Hospital, Mahidol University (COA approval number: MURA2024/529, 30 July 2024) and the Ethics Committee of the National Cancer Institute, Thailand (approval number: 046/2024, 18 October 2024). All participants provided informed consent prior to participation.

### 2.5. Data Collection

After obtaining ethical approval, participants were recruited from the outpatient breast clinic and chemotherapy unit of the National Cancer Institute, Thailand, during routine follow-up visits. Eligible participants were identified by clinic staff and approached by the researcher, who explained the study objectives, procedures, participants’ rights, and obtained written informed consent. Data were collected using self-administered questionnaires completed onsite in a private setting. Completed questionnaires were anonymized using unique codes and securely stored. Participation was voluntary, and clinical care was not affected by study involvement.

### 2.6. Data Analysis

Data were analyzed using the IBM SPSS Statistics software (Version 29.0) [38]. Descriptive statistics summarized participants’ characteristics, FCR, unmet healthcare needs, and QOL. Normality of continuous variables was assessed using skewness/se and kurtosis/se (within ±1.96). Pearson’s product–moment correlation was used to examine the relationship among normally distributed total scores of FCR, unmet healthcare needs, and overall QOL. Spearman’s rank correlation coefficient was used for analyses involving subscale scores that did not meet the normality assumptions, including FCR subscales, unmet healthcare needs domains, and QOL domains. Multiple linear regression analysis was used to examine the effects of FCR and unmet healthcare needs on overall QOL and QOL domains, while controlling for potential confounders, including age, marital status, cancer stage, income, and comorbidities. The assumptions of multiple linear regression models were examined. Residual scatterplots were used to assess normality, linearity, and homoscedasticity for each model. The variance inflation factor (VIF) values for the models did not exceed 10, suggesting no multicollinearity.

## 3. Results

### 3.1. Participant Characteristics

A total of 122 breast cancer survivors were included in the analysis. The mean age of participants was 54.77 years (SD = 9.29). Most participants were married (65.6%) and had completed a bachelor’s degree (34.4%). Clinically, the majority were diagnosed with invasive ductal carcinoma (71.3%), most commonly at stage II (52.4%), and 43.4% had a Luminal A subtype. All participants had undergone surgery; most received chemotherapy (77.9%) and hormonal therapy (94.3%). Detailed demographic and clinical characteristics are presented in Table 1.

### 3.2. Descriptive Statistics of Study Variables

Descriptive statistics for FCR, unmet healthcare needs, and QOL are presented in Table 2. Participants reported a moderate level of FCR (M = 13.39). Unmet healthcare needs were generally low (M = 25.63). Overall QOL was rated as moderate (M = 54.82), with high functional scores (M = 90.16) and low symptom burden (M = 15.53). Similarly, breast-cancer-specific QOL showed moderate-to-high functional scores (M = 72.43) and low symptom scores (M = 9.34).

### 3.3. Associations Among Fear of Cancer Recurrence, Unmet Healthcare Needs, and Quality of Life

As shown in Table 3, FCR was significantly negatively correlated with overall QOL (r = −0.248, *p* < 0.01). Unmet healthcare needs were also negatively correlated with overall QOL (r = −0.261, *p* < 0.01). No significant association was found between FCR and unmet healthcare needs.

At the domain level, FCR was significantly negatively correlated with functional QOL (r = −0.242, *p* < 0.01), whereas unmet healthcare needs were not significantly associated with QOL subdomains or breast-cancer-specific QOL (Table 4). Given the small magnitude of correlations, these findings should be interpreted cautiously.

Multiple linear regression analysis revealed that both FCR and unmet healthcare needs had a negative association with overall QOL (*p* < 0.05) after controlling for variables of age, marital status, cancer stage, income, and comorbidities. However, there was no significant relationship between FCR or unmet healthcare needs and the breast-cancer-specific QOL domain (Table 5).

## 4. Discussion

Breast cancer survivors in this study reported moderate levels of FCR, which indicates manageable concern rather than clinically severe distress. Most participants (76.2%) had stage I–II breast cancer, conditions associated with favorable prognoses and high survival rates [39]. This distribution likely contributed to the moderate FCR levels [40]. Compared with global data, where high FCR prevalence reaches 30–40% of survivors [41,42], the relatively lower distress observed in this study may reflect cultural differences in emotional expression, strong family involvement in caregiving common in Thai society, and accessible follow-up care within tertiary hospital settings [43]. This finding is comparable to the study by Phromcrot et al., which reported a similar moderate level of FCR among breast cancer survivors who had completed primary treatment for at least three months (Mean = 16.70, SD = 6.40) [23]. This interpretation is further supported by Hanprasertpong et al., who found that social support from family, friends, or healthcare providers significantly reduced FCR among Thai breast cancer survivors [20]. Highlighting cultural protective factors increases novelty in comparison with prior Western-focused studies.

Unmet healthcare needs were reported at low levels in the present study, consistent with Pongthavornkamol et al. [19], and may reflect the comprehensive and continuous care typically provided in tertiary hospitals, including coordinated follow-up, symptom monitoring, and access to psychosocial support services. In contrast, Molassiotis et al. [18] reported higher levels of unmet healthcare needs among cancer survivors in Thailand and other Asia–Pacific countries, likely reflecting disparities in healthcare resources, continuity of survivorship care, and access to supportive services across settings. Similarly, Palmer et al. [44] noted that ongoing follow-up and structured supportive care reduced unmet healthcare needs among breast cancer survivors. Beyond individual factors, unmet needs are shaped by structural and social determinants of health, including access to information, psychosocial services, and health literacy. Systemic barriers, misinformation, and cultural taboos have been shown to exacerbate cancer care disparities and adversely affect survivorship outcomes [45,46]. Addressing these factors is essential to reducing unmet needs and improving quality of life.

Overall QOL was moderate, with high functional recovery and low symptom burden. This likely reflects favorable clinical characteristics, as most participants had no distant metastasis and many were classified as having the Luminal A subtype, associated with the best prognosis [7]. Health and global QOL scores were relatively high, indicating good post-treatment recovery and minimal side effects. These findings align with Chua et al. [12], who reported good long-term QOL in breast cancer survivors. By contrast, Paoin et al. [47] found more moderate QOL among survivors undergoing aggressive treatment such as mastectomy and chemotherapy. Differences across studies may reflect differences regarding the timing of follow-up, treatment modalities, and cultural expectations regarding recovery. Functional scales were high, with physical functioning showing the highest scores, suggesting effective rehabilitation and adaptation post-treatment. Symptom score was low, consistent with Fangel et al. [48], who reported low interference of symptoms on QOL after chemotherapy. These results reinforce the need to focus less on physical rehabilitation alone and more on psychosocial survivorship issues such as FCR. In their study, participants reported low symptom interference and good QOL. However, in another study involving breast cancer patients who had undergone radiation therapy within the past year, the role of the functioning dimension score was small [46]. This difference may be attributed to the different treatment modalities and recovery stages.

The negative correlations between FCR, unmet healthcare needs, and QOL highlight the relevance of Mishel’s Uncertainty in Illness Theory [15]. Survivors who experience greater uncertainty—whether triggered by FCR or insufficient healthcare support—are more likely to report lower QOL. Similarly, unmet healthcare needs were negatively correlated with overall QOL [23]. Persistent unmet healthcare needs, such as insufficient information or inadequate follow-up support, can exacerbate uncertainty and diminish QOL [4,49]. Interestingly, no significant association was found between FCR and unmet healthcare needs. This finding may reflect the Thai clinical and cultural context, in which FCR is often perceived as a normative psychological response following cancer treatment. Additionally, participants received comprehensive follow-up care at a tertiary cancer center, which may have reduced the impact of unmet healthcare needs on FCR. From the perspective of Mishel’s Uncertainty in Illness Theory, adequate information and support may have reduced uncertainty, thereby limiting the impact of unmet needs on FCR [43]. Overall, participants reported low levels of unmet healthcare needs, providing context for the lack of a statistically significant correlation with FCR. This contrasts with findings from Korean cancer survivors, among whom unmet needs were positively associated with FCR after treatment completion [50]. Notably, although some associations in the present study were statistically significant, effect sizes were small and should be interpreted cautiously in the clinical context of breast cancer survivorship.

Timely, comprehensive, and continuous care can significantly reduce unmet healthcare needs, alleviate feelings of caregiver-role FCR, and enhance overall QOL for breast cancer survivors. Establishing continuity of care through structured survivorship programs and effective discharge planning is essential for promoting better psychosocial outcomes in this population [51,52]. In addition, discharge planning for breast cancer survivors should be implemented, including clear instructions for symptom management, emotional support, follow-up, and available resources, to enable survivors to return to their daily lives and appropriately care for themselves at home.

### 4.1. Limitations and Future Directions

This study has several limitations. The use of a non-random convenience sample and a cross-sectional design limits causal inference and generalizability. Recruitment from a single tertiary hospital with extensive resources may not represent the experiences of breast cancer survivors in rural or less-resourced settings. Data were collected using self-report questionnaires, which may be subject to recall and social desirability biases, particularly within the Thai cultural context.

Future research should employ longitudinal designs to examine trajectories of FCR, unmet healthcare needs, and QOL across the survivorship continuum. Intervention-based studies targeting psychosocial support and survivorship care planning, particularly among diverse and underserved populations, are needed to inform equitable survivorship care and policy.

### 4.2. Implications

The findings emphasize the importance of integrating survivorship care into routine follow-up. Timely and comprehensive interventions such as structured discharge planning, counseling, and family-inclusive care may reduce both FCR and unmet needs, thereby improving QOL. In resource-rich tertiary hospitals, strategies should focus on psychosocial care, while in resource-limited settings, expanding access to basic survivorship services remains critical. Nurses are ideally positioned to lead survivorship care by systematically screening for FCR, assessing unmet needs, and tailoring interventions to the survivor’s cultural and healthcare context. To further reduce disparities in healthcare and improve outcomes for breast cancer survivors, survivorship programs should consider enhancing access to psychosocial care, providing patient education, and implementing culturally tailored follow-up strategies. Such interventions may help mitigate unmet healthcare needs, reduce FCR, and improve overall QOL among survivors. All recommendations are framed cautiously in line with the correlational nature of the study findings, avoiding overstatement of causality.

## 5. Conclusions

This study demonstrates that FCR and unmet healthcare needs independently and negatively affect QOL among Thai breast cancer survivors following treatment, particularly in functional and psychological aspects. The absence of a significant association between FCR and unmet needs suggests that these constructions may operate independently within this care context. The findings underscore the importance of comprehensive survivorship care that extends beyond disease management to include psychosocial support and individualized follow-up. Incorporating structured survivorship care planning, with attention to symptom management and family involvement, may help improve QOL outcomes. Further longitudinal and mixed-method research is warranted to examine changes in survivorship-related factors over time and to inform the development of contextually appropriate survivorship care strategies.

## Figures and Tables

**Table 1:** Participants’ demographic and clinical characteristics (N = 122).

Characteristics	Frequency	Percentage
Age (years) Mean= 54.77 S.D. = 9.29 Range 30–70 years		
Marital status		
Married	80	65.6
Single	32	26.2
Divorced/Widowed	10	8.2
Educational background		
No formal education	1	0.8
Primary education	27	22.0
Secondary education	29	24.0
Certificate	12	9.8
Bachelor’s degree	42	34.4
Postgraduate education	11	9.0
Occupation		
Homemaker	45	36.9
Daily wage worker	25	20.5
Vendor	11	9.0
State enterprise employee	11	9.0
Retired	10	8.2
Farmer	2	1.6
Government officer	1	10.7
Other	5	4.1
Type of breast cancer		
Carcinoma in situ		
Ductal carcinoma in situ	27	22.2
Lobular carcinoma in situ	1	0.8
Invasive breast cancer		
Invasive ductal carcinoma	87	71.3
Invasive lobular carcinoma	7	5.7
Stage		
1	29	23.8
2	64	52.4
3	29	23.8
Immunohistochemical classification		
Luminal A	53	43.4
Luminal B	41	33.6
HER2-positive	28	23.0
Comorbidities		
No	86	70.5
Yes	36	29.5
Treatment		
Surgery	112	100
Radiation	75	61.5
Chemotherapy	95	79.9
Target therapy	12	9.8
Hormonal therapy		
Tamoxifen	104	85.3
Letrozoles	11	9.0
No	7	5.7

**Table 2:** Possible range, actual range, mean, and standard deviation of fear of cancer recurrence, unmet healthcare needs, and quality of life (N = 122).

Variables	Possible Range	Actual Range	$\bar{X}$	SD
Fear of cancer recurrence	0–36	3–26	13.39	4.53
Unmet healthcare needs	0–105	0.58	25.63	14.82
Overall quality of life	0–100	54.38–55.31	54.82	0.22
Functional scales	0–100	66.67–100	90.16	7.02
Symptom scales	0–100	0–46.5	15.53	10.36
Global health status/quality of life	0–100	41.67–100	80.19	12.14
Breast-cancer-specific quality of life				
Functional scales	0–100	37.5–83.33	72.43	9.54
Symptom scales	0–100	0–44.44	9.34	8.96

**Table 3:** Correlations between fear of cancer recurrence, unmet healthcare needs, and overall quality of life (N = 122).

Variables	Fear of Cancer Recurrence	Unmet Healthcare Needs	Overall Quality of Life
Fear of cancer recurrence	1		
Unmet healthcare needs	0.160	1	
Overall quality of life	−0.248 **	−0.261 **	1

** *p* < 0.01.

**Table 4:** Correlations between fear of cancer recurrence, unmet healthcare needs, and duality of life domains and breast-cancer-specific quality of life domains (N = 122).

Quality of Life Domains	Fear of Cancer Recurrence (r)	Unmet Healthcare Needs (r)
Quality of life		
Functional scales	−0.242 **	0.118
Symptom scales	0.009	0.108
Global health status/quality of life	0.041	0.052
Breast cancer-specific quality of life		
Functional scales	−0.156	−0.054
Symptom scales	0.090	−0.062

** *p* < 0.01.

**Table 5:** Correlations between fear of cancer recurrence and unmet healthcare needs on quality of life using multiple linear regression (N = 122).

Variables	Overall QoL *			Breast-Cancer-Specific QoL					
				Functional Scales **			Symptom Scales **		
	B	S.E.	*p*-Value	B	S.E.	*p*-Value	B	S.E.	*p*-Value
Fear of cancer recurrence	−0.009	0.004	0.042	−0.032	0.194	0.871	0.213	0.192	0.272
Unmet healthcare needs	−0.003	0.001	0.014	0.028	0.058	0.630	−0.005	0.058	0.935
R^2^	0.207			0.132			0.028		
Adjusted R^2^	0.159			0.094			0.014		

B, unstandardized coefficient; S.E., standard errors; R^2^, coefficient of determination. * Model controlling for age, marital status, stage of cancer, income, and comorbidities. ** Model controlling for age, stage of cancer, and comorbidities.

## Data Availability

The original contributions presented in this study are included in the article. Further inquiries can be directed to the corresponding author.

## Abbreviations

The following abbreviations are used in this manuscript:

FCR	Fear of Cancer Recurrence
QOL	Quality of Life
6CIT	6-Item Cognitive Impairment Test—Thai version
FCRI-SF	Fear of Cancer Recurrence Inventory—Short Form
CaSUN	Cancer Survivors’ Unmet Needs Measure
